# Supratentorial Cerebral Arterial Territories for Computed Tomograms: A Mapping Study in 1160 Large Artery Infarcts

**DOI:** 10.1038/s41598-019-48266-2

**Published:** 2019-08-12

**Authors:** Dong-Eog Kim, Jinseong Jang, Dawid Schellingerhout, Wi-Sun Ryu, Jong-Ho Park, Su-Kyoung Lee, Dongmin Kim, Hee-Joon Bae

**Affiliations:** 1 Korean Brain MRI Data Center & Department of Neurology, Dongguk University Ilsan Hospital, Goyang, Korea; 2 AI R&D Center, JLK inspection, Seoul, Korea; 3 Departments of Radiology and Cancer Systems Imaging, University of Texas M.D. Anderson Cancer Center, Houston, USA; 4 Department of Neurology, Myongji Hospital, Hanyang University College of Medicine, Goyang, Korea; 5 Department of Neurology, Seoul National University College of Medicine, Seoul National University Bundang Hospital, Seongnam, Korea

**Keywords:** Stroke, Stroke

## Abstract

We recently generated a high-resolution supratentorial vascular topographic atlas using diffusion-weighed MRI in a population of large artery infarcts. These MRI-based topographic maps are not easily applicable to CT scans, because the standard-reference-lines for axial image orientation (i.e., anterior-posterior commissure line versus orbito-meatal line, respectively) are ‘not parallel’ to each other. Moreover, current, widely-used CT-based vascular topographic diagrams omit demarcation of the inter-territorial border-zones. Thus, we aimed to generate a CT-specific high-resolution atlas, showing the supratentorial cerebrovascular territories and the inter-territorial border-zones in a statistically rigorous way. The diffusion-weighted MRI lesion atlas is based on 1160 patients (67.0 ± 13.3 years old, 53.7% men) with acute (<1-week) cerebral infarction due to significant (>50%) stenosis or occlusion of a single large cerebral artery: anterior, middle, or posterior cerebral artery. We developed a software package enabling the transformation of our MR-based atlas into a re-oriented CT space corresponding to the axial slice orientations used in clinical practice. Infarct volumes are individually mapped to the three vascular territories on the CT template-set, generating brain maps showing the voxelwise frequency of infarct by the affected parent vessel. We then mapped the three vascular territories collectively, generating a dataset of Certainty-Index (CI) maps to reflect the likelihood of a voxel being a member of a specific vascular territory. Border-zones could be defined by using either relative infarct frequencies or CI differences. The topographic vascular territory atlas, revised for CT, will allow for easier and more accurate delineation of arterial territories and borders on CT images.

## Introduction

Cerebral vascular territories are important in stroke research and in the radiologic assessment of infarction, which could guide further investigation and therapeutic decision-making. In a recent study^1^, we generated a high-resolution topographic brain atlas allowing for more accurate delineation of previously defined arterial territories and borders, based on diffusion-weighted (DW) magnetic resonance imaging (MRI) data in 1160 patients with acute intracranial large artery atherosclerotic infarction.

MRI is less widely available than computed tomography (CT) outside of major stroke centers. In addition, about 10% of stroke patients have contraindications or intolerance to MRI^2^. Thus, CT is currently the most commonly used imaging technique for stroke diagnosis worldwide, especially for initial evaluation, even though it is not as sensitive for detection of ischemia as MRI.

**Figure 1:**
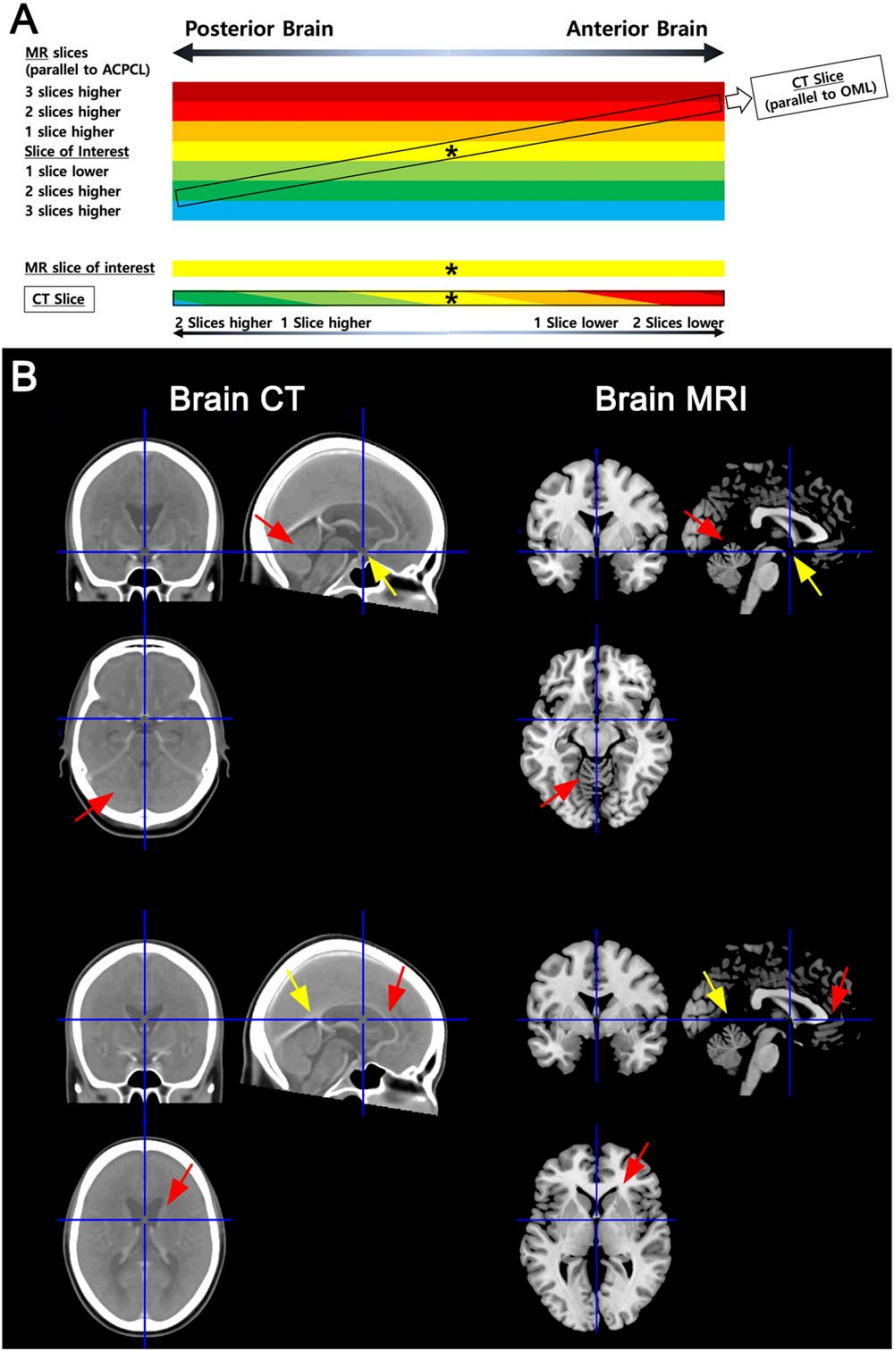
Different slice angles for axial MR vs. CT images. **(A)** The anterior-posterior commissure line (ACPCL) and orbitomeatal line (OML) for the image slicing of respectively MRI and CT are not parallel. Because of the pitch angle difference (average 9°) between the imaging reference lines, the anterior and posterior portions of the axial CT image is respectively about ~10 mm lower and higher in the z-axis, with the central portion overlapping around the rotation point, centered on the MR slice of interest (asterisks). Here, 10-mm difference corresponds to about 2-slice difference, because the slice thicknesses of MRI and CT are respectively 6 mm and 5 mm. **(B)** Anatomical matching of the anterior portions in the MR and CT images (yellow arrows in the upper panel figures) causes a mismatching of the posterior portions (red arrows in the upper panel figures), and vice versa (yellow and red arrows in the lower panel figures).

There are major technical differences in how images are acquired for MR and CT. The standard imaging reference line in clinical brain MRI, defining the plane of axial images, is the anterior / posterior commissure line (ACPCL)^3^, as identified on a mid-sagittal navigator image prior to every study. For CT, the equivalent reference plane is defined by the orbitomeatal line (OML)^4^, a surface reference that connects the outer canthus of the orbit and the center of the external auditory meatus using a laser sight-line on the skin of the patient, prior to each study. These reference planes are close to but “not” identical or parallel to each other, leading to a different angulation and imaging appearance for axial images when comparing MR and (less steep) CT. Because of the pitch angle difference (about 9°)^3^, compared with an axial MR image, the anterior and posterior portions of the axial CT image (given that its central portion is the same as in the MR image) is respectively about ~10 mm lower and higher in the z-axis along the length of the patient’s body. As a result, when using MR-based topographic maps for the determination of vascular territories of stroke lesions ‘within’ a CT slice, and if volumetric data are not available, clinicians perform a mental exercise to deliberately change the corresponding map slices: e.g., moving from the anterior to the posterior direction, positioning 2 slices up, 1 slice up, the same reference slice with its central portion having similar anatomy, and then 1 slice down, and 2 slides down (this applies when the slice thicknesses of the CT scan and MR study are about 5 mm; Fig. 1). CT has relatively poor soft tissue contrast, making the 3-dimensional mental mapping using anatomic features between the modalities relatively difficult.

More importantly, a key feature of vascular territory mapping, the border-zones between territories, seem to not be explicitly indicated on the most widely-used CT-based vascular topographic ‘diagrams’ drawn by Savoiardo^5^ unlike the clear demarcations that we recently delineated in our MRI-based topography study^1^.

The aim of this study is to generate CT-specific high-resolution supratentorial cerebrovascular territorial maps that also mark the border-zones between territories. To this purpose, we developed and utilized a software package that allows the back-and-forth 3D transformation of imaging data from MR to CT, allowing our MR based atlas maps to be projected into CT imaging space, maintaining the normal CT slice orientation and anatomy expected in current clinical practice.

## Methods

As previously described, this is a multicenter study that involved 11 academic stroke centers in Korea^1,6,7^. From May 2011 to February 2013, 12721 patients who were admitted with acute ischemic stroke underwent diffusion-weighted MR imaging (DW-MRI): b-values 0 and 1,000 s/mm^2^, repetition time 2,400 to 9,000 ms, echo time 50 to 99 ms, voxel size 1 × 1 × 3 to 1 × 1 × 5 mm^3^, interslice gap 0 to 2 mm, and thickness 3 to 7 mm. Among these patients, we finally recruited 1160 patients who had supratentorial large artery atherosclerosis (LAA) infarction (on 1.5-T DW-MRI [n = 995] or 3.0-T DW-MRI [n = 165]) due to significant (>50%) stenosis or occlusion of a single large cerebral artery on MR angiography (n = 11604), CT angiography (n = 4844), or conventional angiography (n = 1786), which was assessed by experienced neurologists along with the neuroradiological reports in each participating center: anterior (n = 71), middle (n = 896), or posterior (n = 193) cerebral artery (ACA, MCA, or PCA, respectively). The gold standard used was the consensus of the radiology report and clinical input regarding which parent vessel was responsible for the infarct. When the radiology report and clinical neurological assessment were inconsistent, a dialogue to achieve a consensus was followed to form a final conclusion used for data-capturing purposes. These 1160 patients did not have significant proximal stenosis or occlusion of the intracranial and extracranial carotid arteries that could have given rise to ACA vs. MCA ambiguity in the infarct attribution, and were chosen because a clear link between infarct territory and vascular pathology could be shown in every case. It is notable that additional 77 patients who also met the above angiographic criteria were excluded, because they had multiple territorial infarcts, as determined by the consensus of experienced neurologists in each participating center and a subsequent review by D.-E. Kim (the principal investigator who has a longstanding career in neuroradiology research) using Tatu *et al*.'s atlas^8^. The studies were approved by the ethics committee of the Dongguk University Ilsan Hospital. All patients or their legally authorized representatives provided a written informed consent in accordance with institutional requirements and the Declaration of Helsinki Principles. All methods were performed in accordance with relevant guidelines and regulations.

As previously reported^1,9,10^, high–signal intensity lesions on DW-MR images were semi-automatically segmented and registered onto a standardized volumetric (Ch2better MNI) template set, under meticulous supervision by W.-S. Ryu. The registered binary images for acute infarcts in the ACA, MCA, and PCA group of patients were used to map the three vascular territories individually by generating lesion accumulation maps that show the frequency of infarct for each voxel by the affected parent vessel. These datasets were merged to create a final set of volumetric figures. We then mapped the three vascular territories collectively^1^, generating a dataset of Certainty-Index (CI) maps to show in each voxel the fraction of ACA, MCA or PCA related infarcts as a fraction of the total large territory infarct incidence (defined as the sum of the three territory frequencies).

**Figure 2:**
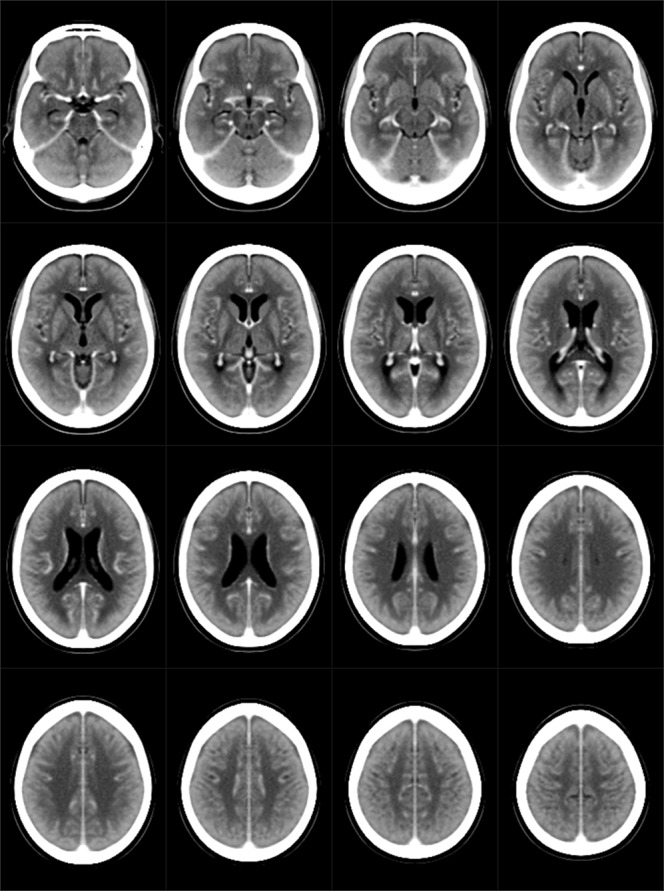
A CT template set for the topographic mapping. The original stroke-control CT template^11^, with the axial slicing angle being in line with the anterior-posterior commissure line, was realigned so that they become parallel to the orbito-meatal line.

Next, we developed a MATLAB (Natik, Massachusetts) software package that 1) adjusts for the pitch angular difference between the ACPCL and OML by rotating (around the anterior commissure) and re-slicing the DW-MRI lesion mapping data on the Ch2better MNI template set (301 × 370 voxels per slice; voxel size = 0.5 mm × 0.5 mm × 6 mm), and 2) registered the realigned lesion data onto a CT template set (Fig. 2; 181 × 243 voxels per slice, voxel size = 1 mm × 1 mm × 5 mm) that was newly prepared by realigning the originally ACPCL-aligned stroke-control CT template^11^ so that it become parallel to the OML. The right hemispheric brain mapping was depicted on the left side of the figures.

## Results and Discussion

**Figure 3:**
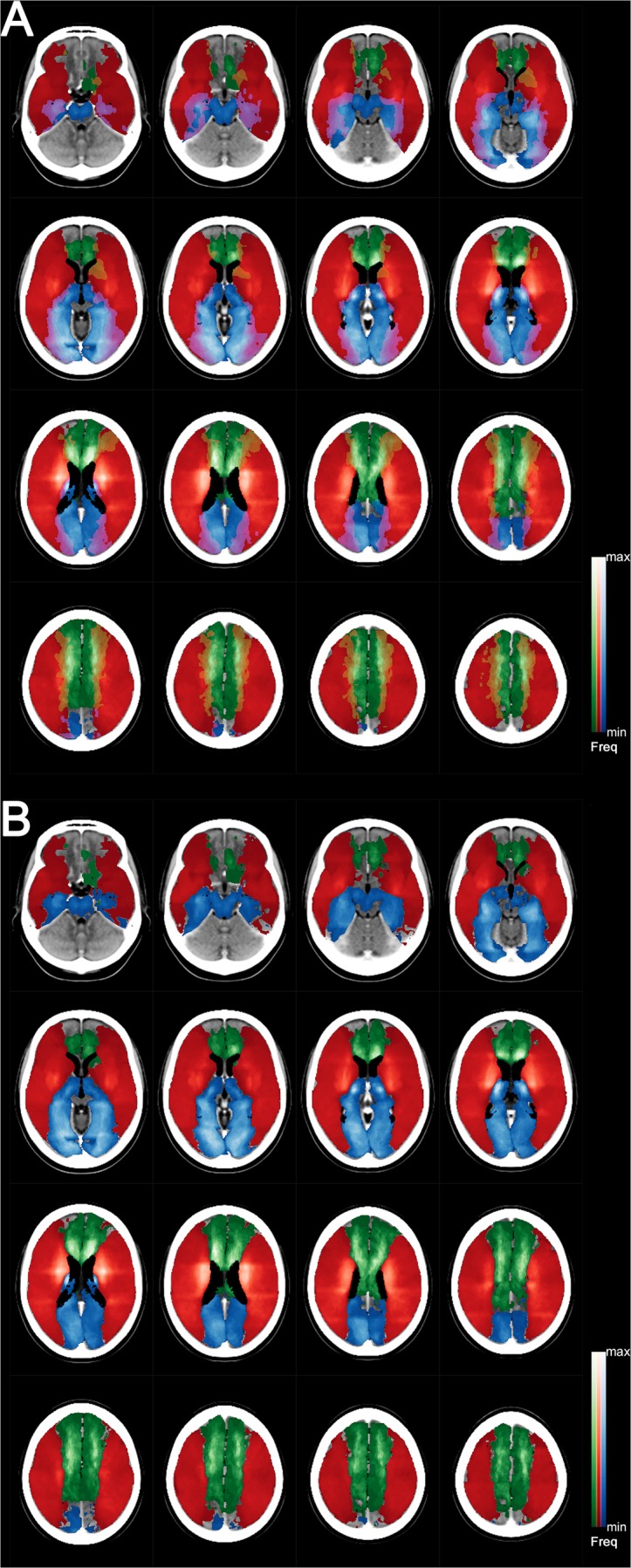
Infarct frequency maps with (**A)** or without **(B)** interterritorial overlaps displayed as color blends. Please see the main text for detailed information.

**Figure 4:**
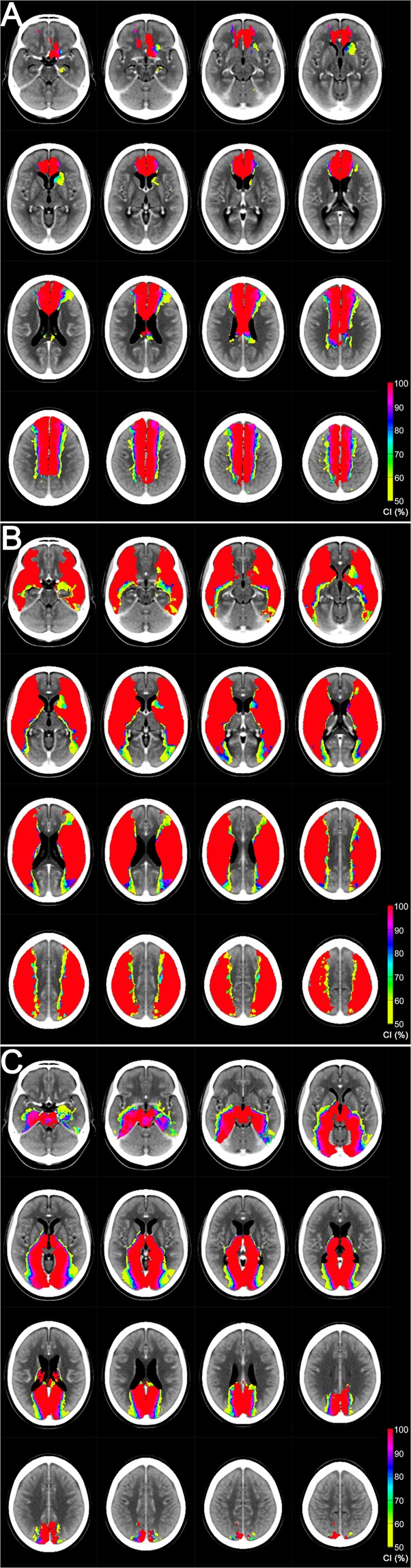
Certainty Index maps for the anterior **(A)**, middle **(B)**, and posterior **(C**) cerebral artery territory. Please see the main text for detailed information.

**Figure 5:**
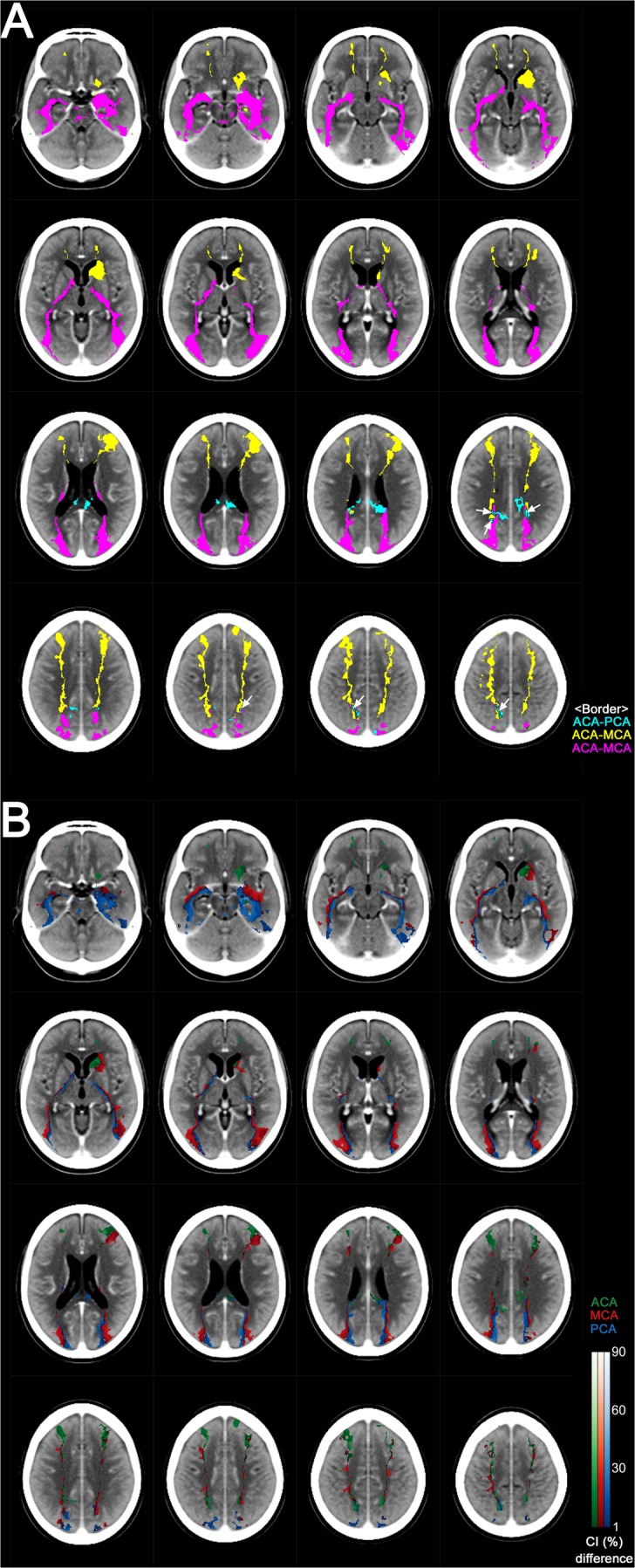
Interterritorial border-zone maps **(A)** and border-line maps **(B)**. Please see the main text for detailed information. Arrows indicate the ACA – MCA – PCA triple border-zone (white areas outlined in black).

Figures 3−5 show the supratentorial vascular territorial maps for CT images using typical CT slice orientation. These maps were generated by the ACPCL-to-OML pitch angle realigning-related reconstruction of the original DW-MR lesion mapping data of 1160 acute stroke patients (67.0 ± 13.3 years old; 623 males) with supratentorial infarction attributable to stenosis or occlusion of either the ACA, MCA, or PCA. Our study confirms the supratentorial arterial boundaries that are depicted in Savoiardo’s vascular territorial diagrams^4^ based on CT studies of infarcts^5^. Unlike the cartoon diagrams, our maps also show border-zones and border-lines, which are represented as overlapping lesion areas with relative lesion frequencies or CI differences displayed.

Figure 3A shows voxelwise lesion frequency heatmaps^1^. Maximum frequencies are 23.9% (17 of 71 patients) for the ACA (green), 14.6% (131 of 896 patients) for the MCA (red), and 17.6% (34 of 193 patients) for the PCA (blue). Color blending was performed for inter-territorial overlap areas to account for blurring between territories. Figure 3B is a modified version, which was generated by allocating each voxel in the overlapping areas in Fig. 3A unequivocally as the territory of a cerebral artery associated with the highest infarct frequency on the voxel (no blurring allowed). The few voxels with equal or near equal frequencies were not color-coded.

Figure 4 shows CI maps for the ACA, MCA, and PCA^1^. CI reflects the likelihood of a voxel being a member of a specific vascular territory vs. the other territories: either ACA, MCA, or PCA infarct frequency divided by total infarct frequency in each voxel.

Figure 5A shows the location of the inter-territorial border-zones^1^. Vascular territorial overlaps are depicted, as derived from the infarct frequency maps in Fig. 3A. Yellow, purple, and sky-blue areas respectively indicate the ACA – MCA, MCA – PCA, and ACA – PCA border-zones. Each voxel in the border-zones has an infarct frequency higher than zero for at least two of ACA, MCA, or PCA infarction. We empirically defined near equality as a frequency difference (i.e., the difference between overlapping territorial values) of each voxel not to exceed 10% (=2.39%) of the highest value (23.9%) of infarct frequencies in every supratentorial voxel for all vascular territories. White areas outlined in black to increase conspicuity are assigned to indicate the special case of the ACA – MCA – PCA triple border-zone (Fig. 5A, arrows).

Figure 5B shows inter-territorial ‘line’ maps^1^. These CI-difference maps were generated, starting with the same data as in Fig. 5A, by forcing a choice for each voxel to belong to only one parent vessel (the one with the highest CI), and color coding it appropriately: green (ACA), red (MCA), and blue (PCA). This reduces the border-zones of Fig. 5A to lines defined by color transitions between territories. Such ‘lines’ are easier to describe and draw, than zones of transition, but should be interpreted as an abstraction of the wider true transition zone, and not as a precise clinical entity^1^. Please note that each interface between different color areas represents a border ‘line’.

In conclusion, to the best of our knowledge, this is the first study that provides CT-specific high-resolution cerebrovascular territorial maps that also display inter-territorial border-zones and border-lines, derived from a large-scale DW-MRI data of 1160 patients with acute intracranial large artery atherosclerotic infarction. The revised topographic brain atlas can be used for objectively defining the supratentorial arterial territories and their borders without (a) the need for volumetric data reformatting and CT-MR image co-registration that allows for the use of an MRI-specific topographic atlas or (b) the need to mentally (and subjectively) perform conversion between MR and CT topographic maps. Further studies are required to validate the CT-specific topographic brain atlas.

## Data Availability

The data generated and/or evaluated in the current study are available from the corresponding author on request.

## References

[1] Kim DE (2019). Mapping the Supratentorial Cerebral Arterial Territories Using 1160 Large Artery Infarcts. JAMA Neurol.

[2] Kim BJ (2014). Current status of acute stroke management in Korea: a report on a multicenter, comprehensive acute stroke registry. Int J Stroke.

[3] Weiss KL (2003). Clinical brain MR imaging prescriptions in Talairach space: technologist- and computer-driven methods. AJNR Am J Neuroradiol.

[4] Yeoman LJ, Howarth L, Britten A, Cotterill A, Adam EJ (1992). Gantry angulation in brain CT: dosage implications, effect on posterior fossa artifacts, and current international practice. Radiology.

[5] Savoiardo M (1986). The vascular territories of the carotid and vertebrobasilar systems. Diagrams based on CT studies of infarcts. Ital J Neurol Sci.

[6] Ryu WS (2017). Stroke outcomes are worse with larger leukoaraiosis volumes. Brain.

[7] Ryu WS (2014). Grading and interpretation of white matter hyperintensities using statistical maps. Stroke.

[8] Tatu L, Moulin T, Vuillier F, Bogousslavsky J (2012). Arterial territories of the human brain. Front Neurol Neurosci.

[9] Kim DE (2019). Estimation of Acute Infarct Volume with Reference Maps: A Simple Visual Tool for Decision Making in Thrombectomy Cases. J Stroke.

[10] Ryu WS (2018). Hemispheric Asymmetry of White Matter Hyperintensity in Association With Lacunar Infarction. J Am Heart Assoc.

[11] Rorden C, Bonilha L, Fridriksson J, Bender B, Karnath HO (2012). Age-specific CT and MRI templates for spatial normalization. Neuroimage.

